# Plasma tenofovir trough concentrations are associated with renal dysfunction in Japanese patients with HIV infection: a retrospective cohort study

**DOI:** 10.1186/s40780-016-0056-5

**Published:** 2016-09-22

**Authors:** Yusuke Kunimoto, Hiroshi Ikeda, Satoshi Fujii, Manabu Kitagawa, Kieko Yamazaki, Hiromasa Nakata, Norimasa Noda, Tadao Ishida, Atsushi Miyamoto

**Affiliations:** 1 Department of Hospital Pharmacy, Sapporo Medical University Hospital, South 1, West 16, Chuo-ku, Sapporo, Hokkaido 060-8543 Japan; 2 Department of Gastroenterology, Rheumatology and Clinical Immunology, Sapporo Medical University School of Medicine, South 1, West 16, Chuo-ku, Sapporo, Hokkaido 060-8543 Japan; 3 Department of Hematology, Japanese Red Cross Medical Center, 4-1-22 Hiroo, Shibuya-ku, Tokyo, 150-8935 Japan

**Keywords:** Combination antiretroviral therapy, HIV, Plasma tenofovir trough concentration, Renal dysfunction, Tenofovir disoproxil fumarate, Therapeutic drug monitoring

## Abstract

**Background:**

Plasma tenofovir (TFV) trough concentrations may be relevant for tenofovir disoproxil fumarate (TDF)-induced renal dysfunction. The purpose of this study was to determine the association between plasma TFV trough concentrations and TDF-induced renal dysfunction in Japanese patients with human immunodeficiency virus (HIV) infection.

**Methods:**

A 48-week, retrospective cohort study was performed with Japanese patients with HIV infection who started a TDF-containing combination antiretroviral therapy regimen. Plasma TFV trough concentrations were obtained at steady state. The following variables were included in the analysis: sex, age, body weight, body mass index (BMI), serum creatinine, CD4+ cell count, HIV-RNA, concomitant medications, comorbidities, plasma TFV trough concentrations, and estimated glomerular filtration rate (eGFR). For comparisons of variables, we used Mann-Whitney U tests or Fisher’s exact tests. Then, variables associated with renal dysfunction in the univariate analysis were entered into correlation analysis.

**Results:**

The analysis included 11 patients. The rate of decrease in eGFR was significantly correlated with body weight (Spearman correlation = −0.645, *p* = 0.041), BMI (Spearman correlation = −0.682, *p* = 0.031), and plasma TFV trough concentrations (Spearman correlation = 0.709, *p* = 0.025).

**Conclusions:**

Despite the small sample size, our findings suggest that higher plasma TFV trough concentrations may cause TDF-induced renal dysfunction. To prevent TDF-induced renal dysfunction, we propose that individual monitoring of plasma TFV trough concentrations should be performed in Japanese patients with HIV infection.

**Electronic supplementary material:**

The online version of this article (doi:10.1186/s40780-016-0056-5) contains supplementary material, which is available to authorized users.

## Background

Tenofovir disoproxil fumarate (TDF) belongs to the nucleotide reverse transcriptase inhibitor (NRTI) class of antiretroviral drugs (ARVs) and is a preferred component of first-line regimens in the US Department of Health and Human Services guidelines [1]. Therefore, promoting safe use of TDF is important during the management of long-term combination antiretroviral therapy (cART). Although TDF has a good safety profile, TDF-induced renal dysfunction is a well-known adverse effect [2, 3].

The most notable adverse effect with TDF is renal dysfunction, and one study reported that the incidence of TDF-induced renal dysfunction was high in Japanese patients [4]. TDF is rapidly converted to tenofovir (TFV) following absorption, and plasma TFV trough concentrations may be relevant for TDF-induced renal dysfunction. High plasma TFV trough concentrations are reportedly associated with a decrease in glomerular function [5]. Furthermore, several studies conducted in Japan reported that low body weight and genetic polymorphisms of drug transporter genes are significantly associated with TDF-induced renal dysfunction [4, 6]. However, reports of the association between plasma TFV trough concentrations and TDF-induced renal dysfunction in Japanese patients with human immunodeficiency virus (HIV) infection are lacking. Moreover, the effect of plasma TFV trough concentrations on renal dysfunction is not guaranteed to be the same between Japanese patients and patients of other ethnic groups. For safe use of TDF in Japan, the risk factors for TDF-induced renal dysfunction in Japanese patients need to be understood.

Therapeutic drug monitoring (TDM) is an important clinical technique for personalized medicine. In ART, TDM is recommended in specific clinical scenarios, such as for cases in which pathophysiological changes (e.g., renal dysfunction) and/or drug-drug interactions adversely affect pharmacokinetics [1, 7]. However, TDM for cART is not recommended for routine use in the clinical care of HIV-infected patients because of a lack of large prospective studies showing that TDM improves clinical or virologic outcomes [1]. We anticipated that clarifying the usefulness of monitoring plasma TFV trough concentrations could prevent TDF-induced renal dysfunction.

Therefore, the purpose of this study was to determine the association between plasma TFV trough concentrations and TDF-induced renal dysfunction.

## Methods

### Patients

A 48-week, retrospective cohort study was performed in Sapporo Medical University Hospital. Data for HIV-infected patients who started a TDF-containing cART regimen between August 2008 and October 2013 were collected from the medical records. Patients who received cART with TDF for at least 2 weeks were enrolled in this exploratory study. Patients with renal disease that could affect renal function were excluded from the analysis. All the patients provided written informed consent before enrollment, and the protocol was approved (25–152) by the ethical review board of Sapporo Medical University.

### Measurements

Patients with plasma TFV trough concentration measurements performed at steady state ≥21 h after drug intake were included in the analysis. Information about the time of last drug intake was reported at the time of sample collection. Pre-dose blood samples were collected in heparinized tubes and centrifuged for 10 min at 3000 rpm; the resultant plasma was removed and stored at −20 °C until analysis. Plasma TFV concentrations were analyzed using high-performance liquid chromatography, and fluorescence was detected at 254 nm for excitation and 425 nm for emission (BML Inc., Japan). Estimated glomerular filtration rate (eGFR) was calculated using the Japanese equation that was developed by the Japanese Society of Nephrology, eGFR = 194 × [serum creatinine]^-1.094^ × [age]^-0.287^ × [0.739 for women] [8]. Renal function was classified as follows: stage 1, eGFR ≥90 mL/min · 1.73 m^2^; stage 2, 90 > eGFR ≥ 60 mL/min · 1.73 m^2^; stage 3, 60 > eGFR ≥ 30 mL/min · 1.73 m^2^; stage 4, 30 > eGFR ≥ 15 mL/min · 1.73 m^2^; and stage 5, eGFR <15 mL/min · 1.73 m^2^ [9]. Renal dysfunction was defined based on the progression of classified chronic kidney disease (CKD) stages after treatment [10]. The rate of decrease in eGFR was defined as the maximum rate of decrease in eGFR from baseline during the study period.

Other variables included in the analysis were sex, age, body weight, body mass index (BMI), body surface area (BSA), serum creatinine concentration, CD4+ cell count, HIV-RNA, concomitant medications, comorbidities, and plasma TFV trough concentrations.

### Statistical analysis

The time from baseline to renal dysfunction was analyzed using the Kaplan-Meier method. Baseline characteristics were compared between patients with and without renal dysfunction using Mann-Whitney U tests or Fisher’s exact tests. Univariate analysis was conducted to reduce the list of potential variables associated with renal dysfunction. The potential variables associated with renal dysfunction with *p* ≤ 0.1 in the univariate analysis were entered into correlation analyses. Statistical significance was defined as a two-sided *p* < 0.05. All analyses were performed using StatMate IV (ATMS Co. Ltd., Tokyo, Japan).

## Results

Of the 12 patients who were enrolled, one patient was excluded due to a congenital solitary kidney. Patient characteristics are presented in Table 1. All of the study patients were men and had a relatively low body weight (median body weight, 56.1 kg). No patient had comorbidities such as hypertension, diabetes, or hepatitis C. The cART regimens included a protease inhibitor for 63.6 % of the patients (*n* = 5, darunavir/ritonavir; *n* = 2, fosamprenavir/ritonavir) and an integrase strand transfer inhibitor (raltegravir) for 36.4 % of the patients. The median time to plasma TFV trough concentration measurements was 9 weeks after starting a TDF-containing cART regimen.

**Table 1:** Patient characteristics at baseline

	Study patients *N* = 11
Male sex, n (%)	11 (100)
Age (years) ^a^	35 (32–45)
Body weight (kg) ^a^	56.1 (51.6–67.3)
BMI (kg/m^2^) ^a^	18.2 (17.6–22.8)
BSA (m^2^)	1.69 (1.60–1.79)
Serum creatinine (mg/dL) ^a^	0.80 (0.70–0.80)
eGFR (mL/min · 1.73 m^2^) ^a^	96.1 (81.9–103.1)
CKD stage, n (%)	
1	7 (63.6)
2	4 (36.4)
3	0 (0)
4	0 (0)
5	0 (0)
CD4^+^ cell count (/μL) ^a^	119 (37–211)
HIV RNA viral load (log_10_/mL) ^a^	4.83 (4.61–5.85)
Protease inhibitors (ritonavir-boosted), n (%)	7 (63.6)
Protease inhibitors (unboosted), n (%)	0 (0)
NNRTIs, n (%)	0 (0)
INSTIs, n (%)	4 (36.4)
Concurrent use of a nephrotoxic drug, n (%)	4 (36.4)
Hypertension, n (%)	0 (0)
Diabetes, n (%)	0 (0)
Hepatitis B, n (%)	3 (27.3)
Hepatitis C, n (%)	0 (0)
Plasma TFV concentration (ng/mL) ^a^	64.0 (56.0–90.5)

*BMI* body mass index, *BSA* body surface area, *eGFR* estimated glomerular filtration rate, *CKD* chronic kidney disease, *NNRTIs* non-nucleoside reverse-transcriptase inhibitors, *INSTIs* integrase strand transfer inhibitors, *TFV* tenofovir

^a^Values are reported as median (interquartile range)

For the seven patients (63.6 %) in whom renal dysfunction occurred, it occurred within 24 weeks from cART initiation. The cumulative incidence of renal dysfunction in these patients was 9.1, 27.3, 45.5, 54.5, and 63.6 % at 4, 8, 12, 16–20, and 24–48 weeks, respectively. The changes in classified CKD stage after treatment in these patients were from stage 1 to 2, stage 1 to 3, and stage 2 to 3 in 3, 2, and two patients, respectively.

Table 2 compares the baseline characteristics between patients with and without renal dysfunction. The rate of decrease in eGFR was significantly different between the 2 groups (*p* = 0.008). In patients with renal dysfunction, age and plasma TFV trough concentrations tended to be higher, and weight and BMI tended to be lower. Data regarding the 11 patients are given in Additional file 1.

**Table 2:** Characteristics of patients with or without renal dysfunction

	Renal dysfunction patients *N* = 7	Non-renal dysfunction patients *N* = 4	*p* value
Age (years) ^a^	45.0 (38.0–52.0)	29.5 (24.0–35.8)	0.072
Body weight (kg) ^a^	52.2 (49.4–62.9)	60.8 (59.0–68.8)	0.078
BMI (kg/m^2^) ^a^	17.7 (17.2–20.9)	21.3 (20.2–23.8)	0.078
BSA (m^2^) ^a^	1.60 (1.57–1.77)	1.70 (1.69–1.79)	0.169
Serum creatinine (mg/dL) ^a^	0.70 (0.65–0.80)	0.80 (0.78–0.83)	0.163
eGFR (mL/min · 1.73 m^2^) ^a^	96.1 (85.9–102.9)	90.4 (82.7–104.2)	0.925
Rate of decrease in eGFR (%) ^a^	33.2 (24.3–38.2)	11.7 (8.0–12.9)	0.008
CD4^+^ cell count (/μL) ^a^	58 (36–133)	204 (99–320)	0.149
HIV RNA viral load (log_10_/mL) ^a^	4.63 (4.52–5.84)	5.33 (4.82–5.92)	0.395
Protease inhibitors (ritonavir-boosted), n (%)	4 (57.1)	3 (75.0)	1.000
INSTIs, n (%)	3 (42.9)	1 (25.0)	1.000
Concurrent use of a nephrotoxic drug, n (%)	2 (28.6)	2 (50)	0.576
Hepatitis B, n (%)	3 (42.6)	0 (0)	0.236
Plasma TFV concentration (ng/mL) ^a^	88.0 (67.0–102.5)	56.0 (50.5–60.5)	0.073

*BMI* body mass index, *BSA* body surface area, *eGFR* estimated glomerular filtration rate, *INSTIs* integrase strand transfer inhibitors, *TFV* tenofovir

^a^Values are reported as median (interquartile range)

**Fig. 1:**
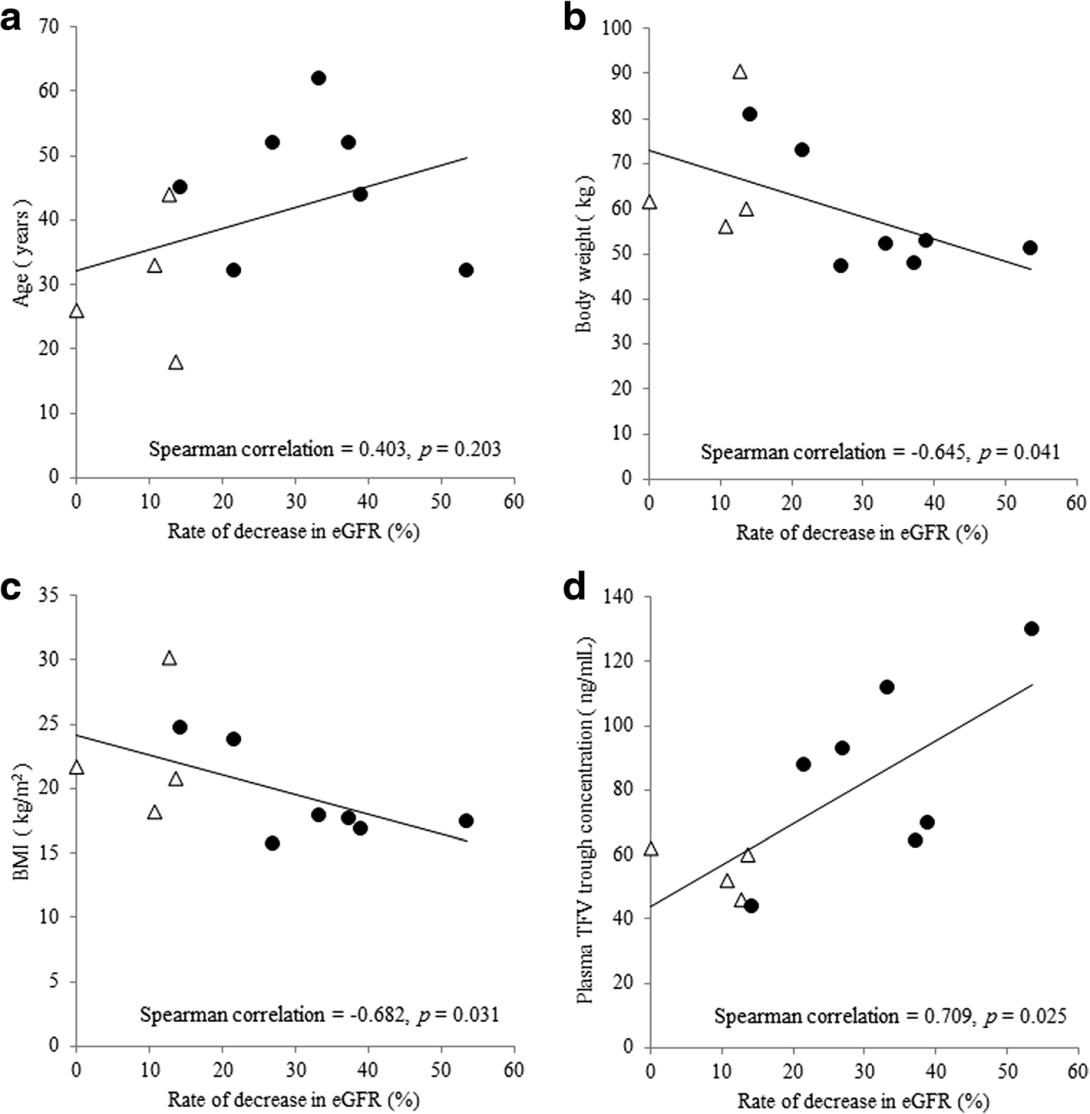
Correlation analysis between eGFR decrease rate and potential variables associated with renal dysfunction. Patients with renal dysfunction are indicated by black circles, and patients without renal dysfunction are indicated by white triangles. eGFR, estimated glomerular filtration rate; BMI, body mass index; TFV, tenofovir

The rate of decrease in eGFR was not significantly correlated with age (Fig. 1a, Spearman correlation = 0.403, *p* = 0.203), but was significantly correlated with weight (Fig. 1b, Spearman correlation = −0.645, *p* = 0.041), BMI (Fig. 1c, Spearman correlation = −0.682, *p* = 0.031), and plasma TFV trough concentrations (Fig. 1d, Spearman correlation = 0.709, *p* = 0.025).

## Discussion

We showed that higher plasma TFV trough concentrations tended to be associated with a decrease in eGFR after starting cART. In principle, ARVs are required for a person’s entire lifetime. Most of the NRTI class of ARVs that are used as first-line regimens are of the renal excretion type. Therefore, choosing ARVs for patients with renal dysfunction is often difficult. Based on our results, we propose individual monitoring of plasma TFV trough concentrations in Japanese patients to prevent TDF-induced renal dysfunction.

Our data indicated that special attention should be given to TDF-induced renal dysfunction within 24 weeks from cART initiation. Not only did renal dysfunction develop within 24 weeks for all 7 patients in the present study, eGFR rapidly decreased in a group administered TDF during the first 24 weeks in a previous study [3]. Therefore, monitoring of renal function should be primarily conducted during the first 24 weeks after initiating a TDF-containing cART regimen.

Plasma TFV trough concentrations showed a stronger correlation with the rate of decrease in eGFR than the other variables, suggesting that plasma TFV trough concentrations are useful to predict the occurrence of TDF-induced renal dysfunction. Additionally, the rate of decrease in eGFR uncorrected for BSA was also significantly correlated with the plasma TFV trough concentration (Spearman correlation = 0.709, *p* ≤ 0.025). Furthermore, in the analysis of the data for the seven patients without concurrent use of a nephrotoxic drug, the rate of decrease in eGFR was significantly correlated with plasma TFV trough concentrations (Spearman correlation = 0.900, *p* < 0.011). The significant correlation between the rate of decrease in eGFR and plasma TFV trough concentrations supports the findings of a previous study in which high plasma TFV trough concentrations were associated with a decrease in eGFR [5]; the median decrease in eGFR at 12 months after cART initiation was 8.5 mL/min in the group with high plasma TFV trough concentrations (>90 ng/mL). In the present study, three patients had high plasma TFV trough concentrations (>90 ng/mL), with decreases in eGFR at 48 weeks after cART initiation of 22.3, 29.4, and 57.2 mL/min. Therefore, the impact of higher plasma TFV trough concentrations on eGFR is greater in Japanese patients. In addition, the occurrence of TDF-induced renal dysfunction might be predicted from higher plasma TFV trough concentrations in Japanese patients. The rate of decrease in eGFR and body weight or BMI was also significantly and negatively correlated, suggesting that the development of TDF-induced renal dysfunction is affected by low body weight and BMI. Low body weight was also associated with TDF-induced renal dysfunction in a previous report [4], and plasma TFV concentrations can be affected by low body weight, genetic polymorphisms of drug transporter genes, and other factors [11]. Additionally, in a study with women, decreasing BMI and other factors were associated with elevated TFV exposure [12]. When measurement of plasma TFV trough concentrations for individual monitoring purposes in Japanese patients is not possible, we suggest that low body weight or low BMI could serve as a reasonable alternative predictor of TDF-induced renal dysfunction.

Our study has several limitations. First, the main limitation of our study is the small sample size. Future studies are needed to replicate our results with a larger sample size. Second, formulations containing TDF are only available in oral tablets, and powder formulations for the purposes of dose adjustment do not exist in Japan. To reduce the single dose amount and extend the dosing interval (e.g., every 48 h), crushed tablets could be used to control higher plasma TFV trough concentrations. However, this could cause issues with maintaining medication adherence. Therefore, reducing the dose of TDF based on TDM is not currently easy.

## Conclusions

In conclusion, this study showed that higher plasma TFV trough concentrations are associated with a decrease in eGFR in Japanese patients, and plasma TFV trough concentrations might predict the occurrence of TDF-induced renal dysfunction. In the future, to prevent TDF-induced renal dysfunction, reduction of the TDF dose or a switch from TDF to a different ARV could be considered with high plasma TFV trough concentrations. Although more studies are needed to identify the factors related with TDF-induced renal dysfunction, we believe that the use of plasma TFV trough concentrations could help prevent this adverse event.

## Additional file

Additional file 1 Data regarding the 11 patients. (XLSX 10 kb)

## Abbreviations

ARV: Antiretroviral drug
BMI: Body mass index
cART: Combination antiretroviral therapy
eGFR: Estimated glomerular filtration rate
HIV: Human immunodeficiency virus
NRTI: Nucleotide reverse transcriptase inhibitor
TDF: Tenofovir disoproxil fumarate
TDM: Therapeutic drug monitoring
TFV: Tenofovir
